# The effector protein ExoY secreted by *Pseudomonas aeruginosa* is a nucleotidyl cyclase with preference for GTP

**DOI:** 10.1186/1471-2210-11-S1-P74

**Published:** 2011-08-01

**Authors:** Ulrike Voigt, Heike Burhenne, Lena Sonnow, Christine Wölfel, Corinna Spangler, Daniel Ladant, Dara W Frank, Volkhard Kaever, Roland Seifert

**Affiliations:** 1 Institute of Pharmacology, Medical School of Hannover, D-30625 Hannover, Germany; 2 Institut Pasteur, Department of Structural Biology and Chemistry, F-75724 Paris, France; 3 Department of Microbiology and Molecular Genetics, Medical College of Wisconsin, Milwaukee, WI 53326, USA

## Background

*Pseudomonas aeruginosa* is an important opportunistic pathogen causing serious pulmonary, urogenital and systemic infections. *P. aeruginosa* injects four effector proteins into host cells *via* the type III secretion system. ExoY is one of these proteins.

ExoY was originally classified as adenylyl cyclase with homology to the typical calmodulin-stimulated adenylyl cyclase exotoxins CyaA from *Bordetella pertussis* and edema factor from *Bacillus anthracis*, but the pathophysiologial function of ExoY has remained elusive [1,2]. Recently, our group showed that CyaA and edema factor possess a rather broad base specifity (ATP >> CTP > UTP) [3,4], raising the question of wether ExoY may also bind and metabolize nucleoside 5’- triphosphates other than ATP.

## Methods

We determined cyclic nucleotide concentrations in cells transfected with ExoY plasmid or infected with *P. aeruginosa* with a highly sensitive HPLC-MS/MS method. Moreover, we determined the catalytic activity of purified ExoY.

## Results

In mammalian cells transfected with ExoY plasmid and infected with ExoY-encoding *P. aeruginosa*, massive production of cGMP and cUMP was observed, with little production of cAMP. Purified ExoY was a highly effective nucleotidyl cyclase with the substrate preference GTP >> UTP ~ ATP > CTP. Fluorescence resonance energy transfer studies with methylanthraniloyl-substituted nucleotides corroborated the preference of ExoY for GTP. In contrast to ExoY, CyaA induced accumulation of cyclic nucleotides in the order cAMP > cCMP > cUMP in mammalian cells, and edema factor induced only cAMP- and cCMP accumulation.

**Table 1:** Comparison of ExoY with CyaA and EF

Parameter	ExoY	CyaA	EF
**Bacterial source**	*Pseudomonas aeruginosa*	*Bordetella pertussis*	*Bacillus anthracis*
**Secretion type**	III	I	II
**Function as toxin**	Unknown, “antitoxin?”	yes	yes
**Activation mechanism**	Cytosolic cofactor (mammalian cells and *D. discoideum*)	calmodulin	calmodulin
**Substrate-specificity purified enzyme**	GTP >> UTP ≥ ATP > CTP	ATP >> CTP > UTP > GTP	ATP >> CTP > UTP > GTP
**Substrate-specificity intact cells**	UTP ~ GTP > ATP > CTP (except for B103 cells)	ATP >> CTP > UTP >> GTP (ineffective)	ATP >> CTP >> UTP and GTP(ineffective)

## Conclusion

ExoY is a nucleotidyl cyclase with preference for GTP, and the substrate-specificity of ExoY is clearly different from that of CyaA and edema factor. Our data open the door for future studies aiming at the elucidation of the as yet unknown pathophysiological function of ExoY and which role cCMP, cGMP and cUMP play in this process.
